# Gastroesophageal reflux disease and oral manifestations

**DOI:** 10.1186/1824-7288-40-S1-A73

**Published:** 2014-08-11

**Authors:** Claudio Romano, Sabrina Cardile

**Affiliations:** 1 Department of Pediatrics, University of Messina, Messina, Italy

## 

Gastroesophageal Reflux (GER) is a common condition in childhood characterized by the rise of gastric contents into the esophagus. According to the International Consensus of the Montreal, gastroesophageal reflux disease (GERD) is defined as "the condition that develops when the retrograde passage of gastric contents causes troublesome symptoms and/or complications that result in an impairment of the quality of life of these patients" [1]. In pediatric population, there are conditions at risk of developing GERD, like neurological impairment, history of esophageal atresia repaired and obesity [2]. The typical symptoms can include heartburn with or without regurgitation. For extra-esophageal syndromes, only dental erosions and Sandifer syndrome are considered conditions related to GERD. Acid reflux at the level of the oral cavity, in fact, can cause the dissolution of the tooth enamel, especially at the level of the palatal surfaces of the back teeth, with a reported prevalence of up to 42% [3]. In a pediatric cross sectional study, in 112 children was found a significant incidence for dental erosion in patients with GERD respect to control group, both in primary and permanent teeth [4]. In general, however, oral manifestations of GERD are reported mainly. In pediatric population, the dental erosion are not considered primary extra-esophageal manifestation of GERD because, when present, can be associated at multiple factors [5]. The typical manifestations can be considered dental caries, dry mouth, feeling at oral acid/burning sensation, halitosis, erythema of the palatal mucosa and uvula. For diagnosis is mandatory exclude other causes, like dietary factors, drugs, poor oral hygiene, eating behavior disorders, genetic and racial factors. The esophageal pH monitoring and/or endoscopy are usually necessary just to confirm the diagnosis of GERD. In this group of patients it’s possible to start a pharmacological therapy in association with modifications of the diet (quantity and frequency of intake of foods and reduction of the beverages that contain fat, sugars and acids) to decrease the time of exposure at gastric acid and secretions. Therapeutic options are based on the medical treatment or surgery in severe cases [6], although there are few studies to evaluate the efficacy of the treatment of GERD to prevent oral cavity lesions.
